# Effect of Redox Modulating NRF2 Activators on Chronic Kidney Disease

**DOI:** 10.3390/molecules190812727

**Published:** 2014-08-20

**Authors:** Bo-hyun Choi, Kyung-Shin Kang, Mi-Kyoung Kwak

**Affiliations:** 1 College of Pharmacy, The Catholic University of Korea, Bucheon, Gyeonggi-do 420-743, Korea; E-Mail: bhchoi37@gmail.com; 2 Daewon Foreign Language High School, Kwangjin-gu, Seoul 143-713, Korea; E-Mail: ks960127@naver.com

**Keywords:** chronic kidney disease, oxidative stress, inflammation, NRF2, sulforaphane, resveratrol, curcumin, cinnamic aldehyde

## Abstract

Chronic kidney disease (CKD) is featured by a progressive decline of kidney function and is mainly caused by chronic diseases such as diabetes mellitus and hypertension. CKD is a complex disease due to cardiovascular complications and high morbidity; however, there is no single treatment to improve kidney function in CKD patients. Since biological markers representing oxidative stress are significantly elevated in CKD patients, oxidative stress is receiving attention as a contributing factor to CKD pathology. Nuclear factor erythroid-2 related factor 2 (NRF2) is a predominant transcription factor that regulates the expression of a wide array of genes encoding antioxidant proteins, thiol molecules and their generating enzymes, detoxifying enzymes, and stress response proteins, all of which can counteract inflammatory and oxidative damages. There is considerable experimental evidence suggesting that NRF2 signaling plays a protective role in renal injuries that are caused by various pathologic conditions. In addition, impaired NRF2 activity and consequent target gene repression have been observed in CKD animals. Therefore, a pharmacological intervention activating NRF2 signaling can be beneficial in protecting against kidney dysfunction in CKD. This review article provides an overview of the role of NRF2 in experimental CKD models and describes current findings on the renoprotective effects of naturally occurring NRF2 activators, including sulforaphane, resveratrol, curcumin, and cinnamic aldehyde. These experimental results, coupled with recent clinical experiences with a synthetic triterpenoid, bardoxolone methyl, have brought a light of hope for ameliorating CKD progression by preventing oxidative stress and maintaining cellular redox homeostasis.

## 1. Chronic Kidney Disease (CKD)

An essential function of kidneys is the filtration of excess waste products that build up in the blood. As a result, waste will accumulate in the circulation system if the kidney fails to function properly, eventually progressing to end stage renal disease [1]. Renal failure occurs gradually, as opposed to showing overt, immediate symptoms. While chronic kidney disease (CKD) is being increasingly diagnosed worldwide, treatment methods such as dialysis or transplantation are either too costly or ineffective, so the condition is considered a public health problem that requires significant further research [2]. According to the Kidney Disease Quality Outcome Initiative (K/DOQI), CKD is defined as the atrophy of the kidney or of renal functions [3]. Renal atrophy can be identified in two different cases: when the glomerular filtration rate (GFR) does not reach above 60 mL/min/1.73 m^2^ for more than three months, and when albuminuria, defined as an albumin-to-creatinine ratio above 30 mg/g is present. However, when diagnosing the development and progression of CKD, the increase in urine albumin cannot be a direct marker—instead, it acts as a sign of renal deterioration as albuminuria indicates endothelial dysfunction of kidneys [2].

The progression of renal disease can be separated into five stages, the criterion of which is GFR. Patients in the first stage share similar GFR (≥90) to healthy counterparts (kidney damage with normal or increased GFR), making it difficult to identify the disease according to GFR alone. If kidney disease is suspected at this stage, it is imperative to delay its progression and minimize risk factors. Stage 2 is characterized by a 60–89 mL/min/1.73 m^2^ level of GFR (kidney damage with mild decreased GFR). As in Stage 1, it is difficult to distinguish CKD, but supplementary information such as albuminuria, proteinuria, and hematuria act as complementary indicators of CKD. The GFR of stage 3 is 30–59 mL/min/1.73 m^2^, and it is this stage in which early renal insufficiency is detected (moderately decreased GFR). Additional caution is required as complications like cardiovascular disease may accompany initial kidney damage. Stage 4, or pre-End-Stage Renal Disease (ESRD), has a GFR level of 15–29 mL/min/1.73 m^2^ (severely decreased GFR). Kidney replacement therapy must be considered when the condition worsens, since pharmacotherapy or lifestyle changes are not as effective in this stage. Finally, ESRD is declared upon the fifth stage when the kidney is completely deprived of its function. If transplantation is not available, it is crucial to undergo dialysis at this point [2].

Two main causes of CKD can be attributed to diabetes mellitus (DM) and hypertension [4]. Kidney damage that stems from diabetes is called diabetic nephropathy (DN). It occurs when high blood sugar resulting from diabetes impairs blood vessels in the kidney, an organ densely populated by myriad small vessels, which ultimately leads to severe renal degeneration. Gradual buildup of this damage eventually leads to renal failure, in which the kidney loses its function [5]. Major pathological changes instigated by DN are mesangial cell proliferation, glomerular hypertrophy and sclerosis, accumulation of extracellular matrix (ECM) in the glomerular basement membrane, and end-stage interstitial fibrosis [6,7]. Hypertension is the second leading cause of CKD, succeeding diabetes [8]. Hypertensive nephropathy patients are advised to maintain their blood pressure to 130/80 mm Hg to prevent CKD [9]. Currently, there is no single treatment to improve kidney function in CKD. Approaches to slow down the progression of CKD are limited to normalization of blood pressure blood glucose, and insulin. Therefore, the development of novel therapies to retard or reverse the decline of kidney function is highly needed.

## 2. Oxidative Stress and Inflammation in CKD

### 2.1. Oxidative Stress in CKD

Oxidative stress is produced when reactive oxygen species (ROS) production goes over the limit of the capacity of the antioxidant defense systems of the body [10]. This is a common phenomenon found in CKD lesions, and is considered to play a critical role in both the progression of CKD and related complications [10,11,12]. Over 90% of ROS are produced in the mitochondria during cellular respiration [13]. While a large majority of oxygen is converted into water, one electron reduction of O_2 _produces a “primary” ROS, superoxide (O_2_^−^) [14]. In turn, O_2 _is transformed into hydrogen peroxide (H_2_O_2_) in mitochondria [15]. Yuan *et al.* [16] showed that mitochondrial dysfunction is involved in the pathogenesis of epithelial-mesenchymal transition (EMT) in renal proximal tubular epithelial cells, which is implicated for kidney fibrosis. The unilateral ureteral obstruction (UUO) model performed by Nishida *et al*. [17] demonstrated that mitochondrial proteins, especially in the electron transport complexes, decreased at an early stage of kidney fibrosis. Impaired mitochondrial respiration system and higher levels of oxidative stress markers were observed in CKD patients under hemodialysis treatment [18].

Additional cytosolic sources of ROS, including NADPH oxidase, xanthine oxidase, and lipoxygenases initiate and increase ROS production as well. Indeed, a chronic renal failure model, induced by renal mass reduction, showed up-regulated NAD(P)H oxidase in rats [19]. Fortuno *et al*. [20] reported that increased NADPH oxidase activity results in enhanced superoxide generation in patients with early CKD. Moreover, single nucleotide polymorphism in the coding region of p22phox, a key component of NADPH oxidase, was associated with elevated levels of oxidative stress in the kidney and consequent dialysis requirement [21]. Because the serum uric acid, an important source of oxidative stress in CKD, is produced by xanthine oxidase, blockade of xanthine oxidase led to renoprotective effects in 5/6 nephrectomized rats [22], diabetic mice [23] and UUO rats [24]. The 12/15-lipoxygenase was significantly up-regulated in the renal cortex of high-calorie/high-fat diet fed mice, which is a model of pre-diabetic neuropathy [25]. Another source of ROS production comes from activation of renin-angiotensin system (RAS). Activation of AT1 receptor by angiotensin II administration generated a higher level of superoxide in CKD animals than that of the normal animals [19,26]. A local increase of angiotensin-converting enzyme (ACE) in the kidney was associated with the progress of tubulointerstitial renal injury by hypokalemia, hypertension, and angiotensin II infusion in rats [27].

The level of ROS in the body is rigidly controlled by antioxidant enzymes, including superoxide dismutase (SOD), catalase (CAT), glutathione peroxidase (GPx), and thiol molecules such as glutathione (GSH). High concentration glucose-treated cells produced more ROS than cells with normal glucose concentration. In this condition, overexpression of MnSOD effectively decreased ROS production, preventing related diabetic complications [28]. A study by Hinerfeld *et al*. [29] showed that mitochondrial *sod* null mice developed severe oxidative stress and diabetic complications. On the other hand, transgenic mice with CAT overexpression showed renoprotective effects following streptozotocin (STZ) treatment [30].

### 2.2. Inflammation in CKD

In pathophysiological conditions, oxidative stress and inflammation are inseparable from each other. Hasegawa *et al.* [31] found that levels of tumor necrosis factor-α (TNFα) and interleukin-1 (IL-1) were significantly increased in glomerular basement membranes of STZ-treated diabetic rats. Kidneys from diabetic rats showed higher IL-6 expression along with increased renal cortical IL-6 mRNA. The elevation of cytokines levels was related to the increase in kidney weight and urinary albumin excretion [32]. Additional reports verified that mRNA and protein levels of TNFα are elevated in glomerular cells of diabetic animals [33,34]. Particularly, TNFα increased ROS production in kidney mesangial cells [35], and activated NADPH oxidase causing glomerular injuries [36]. Besides, surface proteins of inflammatory cells, called adhesion molecules, play important roles in diabetic nephropathy. Coimbra *et al*. demonstrated that obese Zucker rats with T2DM developed early podocyte damage and interstitial macrophage infiltration, implying progressive renal disease, and particularly that an increase of intracellular adhesion molecule 1 (ICAM-1) was observed in glomerular and epithelial cells [37]. The level of vascular cell adhesion protein 1 (VCAM-1) is correlated to albuminuria in type 2 diabetic hypertensive patients [38]. Expression of pro-inflammatory cytokines and adhesion molecules, which are major mediators of inflammation, is regulated by transcription factor nuclear factor-κB (NF-κB) [39]. There are lines of evidence indicating that NF-κB activation is associated with oxidative stress-associated renal fibrosis [40,41]. Rats with 5/6 partial nephrectomy were shown to have increased levels of p65, a NF-κB subunit, in glomerular cells [42]. In STZ-induced diabetic rats, NF-κB activation was found in the renal cortex [43]. 

### 2.3. Biological Markers of Oxidative Stress and Inflammation in CKD

In the clinical setting, levels of oxidation markers of lipid, protein, and DNA are heightened in CKD patients (Table 1). Increased malondialdehyde (MDA), a lipid-associated oxidation marker, in CKD patients implies severe glomerulosclerosis [44]. Grone *et al.* [45] demonstrated that levels of hypochlorous acid (HCIO)-modified proteins increase in renal podocytes and glomeruli from CKD patients. Elevation of 8-oxo-7,8-dihydro-2'-deoxyguanosine (8-oxo-dG) was associated with ROS-related DNA damage in CKD patients [46]. Levels of plasma 8-isoprostanes and GFR showed an opposing relationship in CKD patients [47,48]. In addition, although there were some fluctuations depending on performed studies, levels of antioxidant genes such as SOD and CAT, and GSH contents were diminished in CKD patients [49,50,51]. Polymorphism of the *MnSOD* gene was found to be related to the incidence of DN in Chinese, Japanese and Koreans subjects with T2DM [52,53,54]. Moreover, SOD expression levels were positively correlated with GFR [55] and levels of GSH content were also positively associated with renal creatinine clearance [49].

**Table 1:** Biological markers of oxidative stress and inflammation in CKD patients.

Type of Markers	Group	Specific Marker	Refs.
Oxidative markers	Lipid	F2-isoprostanes	[56,57,58]
		Malondialdehyde (MDA)	[59,60]
		Thiobarbituric acid- reactive substance	[61]
	Protein	Carbonyls	[62,63]
		Advanced glycation end-products (AGEs)	[64]
		Advanced oxidation protein products (AOPP)	[65,66]
		Oxidized low density lipoproteins (OxLDL)	[67]
	DNA	8-Oxo-7,8-dihydro-2'-deoxyguanosine (8-oxo-dG)	[46,68]
		DNA strand breaks	[69,70]
Inflammatory markers		C-reactive protein (CRP)	[71,72]
		IL-1	[73]
		IL-6	[72]
		TNFα	[73,74]

In addition to the increase in oxidative markers, CKD patients are constantly exposed to transient infections, comorbidities, and intermittent stimulus of dialysis [75]. As a result, it is generally accepted that pro-inflammatory cytokines such as TNFα and IL-1β are amplified in hemodialysis patients [73,76]. According to Shlipak *et al*. [72], patients with renal insufficiency showed markedly higher levels of C-reactive protein (CRP) and IL-6 than those with normal kidney functions. The study by Oberg *et al*. reaffirmed this finding: inflammatory biomarkers such as CRP and IL-6 were amplified in CKD subjects as compared to healthy control groups [77].

## 3. Involvement of NRF2 Signaling in CKD Pathology

### 3.1. NRF2 as a Crucial Regulator of the Antioxidant Defense System

**Figure 1:**
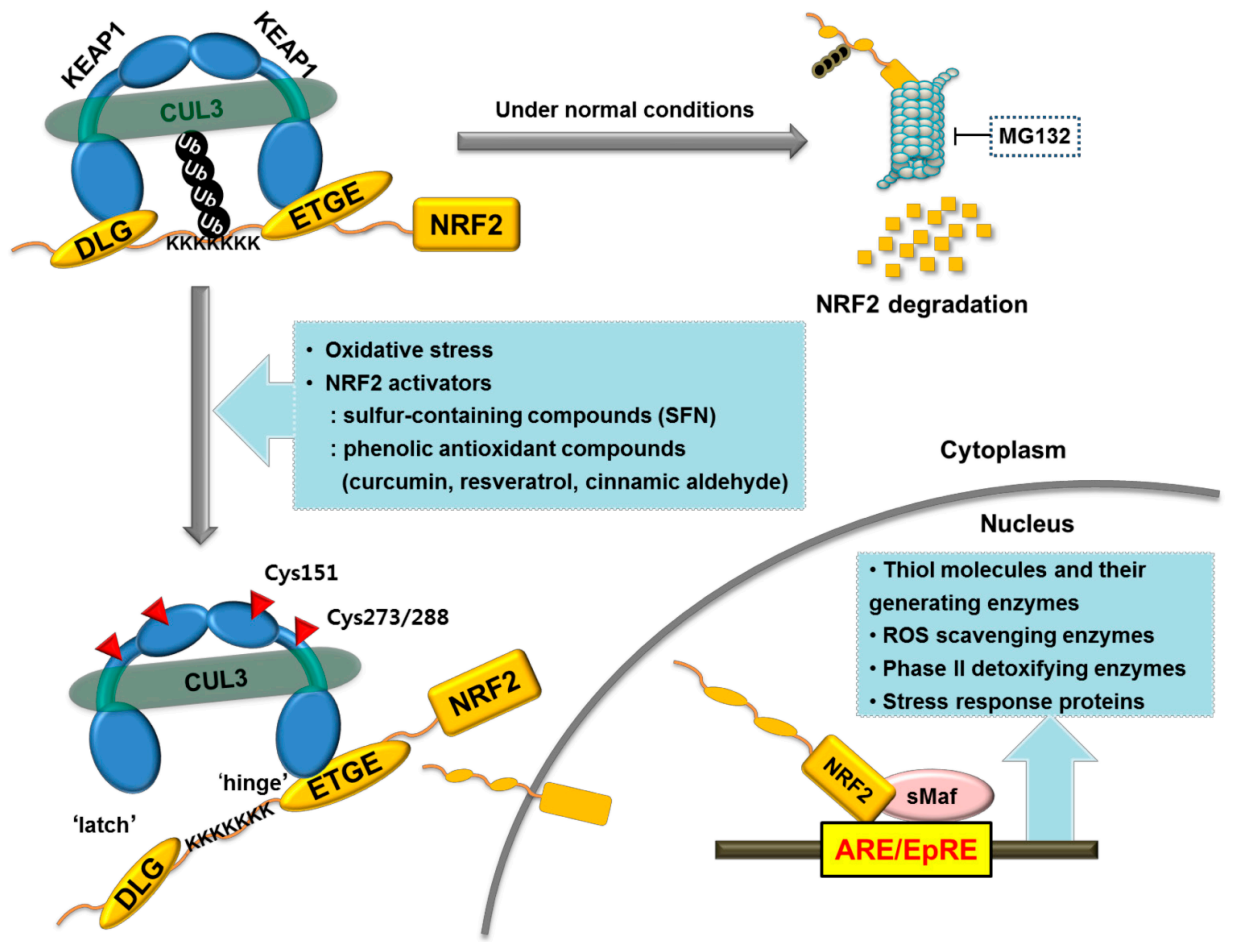
Regulation of antioxidant and detoxifying genes by the NRF2-Keap1 pathway. Under normal conditions, NRF2 is continuously degraded by the KEAP1-Cul3-proteasome axis. When Cys residues of KEAP1 protein are modified by sulfhydryl reactive chemicals, conformational KEAP1 changes lead to NRF2 liberation and transcriptional activation of an array of ARE-bearing genes, encoding detoxifying enzymes, ROS scavenging enzymes, thiol molecules, and their generating enzymes.

Nuclear factor-erythroid 2-related factor 2 (NRF2) is a redox-sensitive transcription factor with the basic leucine zipper (bZIP) motif. Belonging to the cap ‘n’ collar (CNC) family, NRF2 is crucial in regulating the basal and inducible expressions of various antioxidant and cytoprotective genes, which can counteract oxidative and electrophilic stress [78,79]. NRF2 activity is primarily governed by the Kelch-like ECH-associated protein 1 (KEAP1). Under normal conditions, cytosolic protein KEAP1 sequesters NRF2 in the cytoplasm and forwards ubiquitination and subsequent degradation by bridging NRF2 to a Cul3-based E3 ligase. The binding and regulation of KEAP1 and NRF2 have been explained by a “hinge and latch” model: the KEAP1 dimer formed through the BTB domain of this protein interacts with one molecule of NRF2 through conserved motifs DLG and ETGE (Figure 1). In particular, binding affinity of DLG motif of NRF2 is lower than that of ETGE motif; therefore a cysteine-rich protein KEAP1 can sensitively transduce a variety of redox signals to this binding motif. When KEAP1 cysteine residues are modified by ROS or electrophiles, KEAP1 undergoes conformational changes, leading to the dissociation of the DLG motif from KEAP1 and the blockade of ubiquitination/proteasomal degradation of this protein. Then NRF2 can translocate into the nucleus to activate its target gene transcription [80,81]. Within the nucleus, NRF2 heterodimerizes with small Maf proteins and transactivates the expression of genes with the antioxidant response element (ARE), which is also called as the electrophile response elements (EpRE).

The first *in vivo* demonstration of the involvement of NRF2 in detoxifying gene regulation has been reported in 1997: The Yamamoto group showed that the induction of Nqo1 and glutathione *S*-transferase (Gst) by butylated hydroxyanisole (*t*BHA) treatment was largely attenuated in *nrf2* deficient mice [82]. After this observation, numerous studies have identified a wide array of genes as NRF2-target genes [83,84,85,86]. These include genes encoding antioxidant enzymes, phase II detoxification enzymes, NADPH-generating enzymes, drug transporters, metabolism-related enzymes, and proteasome (Table 2). Among antioxidant proteins, NRF2 controls the expression of direct ROS scavenging enzymes such as glutathione peroxidase (GPx) and SOD; GSH generating enzymes such as the catalytic and modifier subunit of γ-glutamate cysteine ligase (GCLC and GCLM, respectively), and glutathione reductase (GSR); and thiol molecules such as thioredoxin. NRF2 is also involved in the expression of NADPH-generating system, which includes malic enzyme 1, glucose-6-phosphate 1-dehydrogenase, and 6-phosphogluconate dehydrogenase. Moreover, NAD(P)H:quinone oxido-reductase 1 (NQO1), heme oxygenase-1 (HO-1) and aldoketo-reductase family are also well-known NRF2 target genes.

**Table 2:** Genes regulated by NRF2 in mouse (m) and human (h).

Functional Classification	Gene Name	Description	Species	
Antioxidant proteins	*GCLC*	γ-Glutamate-cysteine ligase, catalytic subunit	m	h
	*GCLM*	γ-Glutamate-cysteine ligase, modifier subunit	m	h
	*GSR*	Glutathione reductase	m	h
	*GPx1*	Glutathione peroxidase 1 (or 4)	m	
	*GPx2*	Glutathione peroxidase 2	m	h
	*TXNRD*	Thioredoxin reductase	m	h
	*TXN*	Thioredoxin	m	h
	*PRDX1 &* *6*	Peroxiredoxin 1 (or 6)	m	h
	*CAT*	Catalase	m	h
	*SOD*	Superoxide dismutase	m	h
	*SRXN1*	Sulfiredoxin 1	m	h
	*GGT1*	γ-Glutamyltransferase 1		h
	*GLRX*	Glutaredoxin		h
Phase I oxidation, reduction and hydrolysis enzymes	*ALDH3A1*	Aldehyde dehydrogenase 3A1	m	h
	*ADH7*	Alcohol dehydrogenase 7	m	
	*AKR1B1*	Aldo-keto reductase 1B1	m	h
	*AKR1C1*	Aldo-keto reductase 1C1		h
	*CBR1*	Carbonyl reductase 1	m	
	*EPHX1*	Microsomal epoxide hydrolase 1	m	h
	*NQO1*	NAD(P)H:quinone oxidoreductase 1	m	h
	*CYP2B9*	Cytochrome p450, 2B9		
Phase II detoxifying enzymes	*GSTM1*	Glutathione *S*-transferase class mu 1 (or 2,4,5,6)	m	
	*GSTM3*	Glutathione *S*-transferase class mu 3	m	h
	*GSTA1*	Glutathione *S*-transferase class alpha 1 (or 2,3,4)	m	
	*MGST1*	Microsomal glutathione *S*-transferase 2	m	h
	*MGST2*	Microsomal glutathione *S*-transferase 3	m	
	*UGT1A6*	UDP glucuronosyltransferase 1A6		h
	*UGT2B1*	UDP glucuronosyltransferase 2B1	m	
	*UGT2B5*	UDP glucuronosyltransferase 2B5	m	
NADPH-generating enzymes	*ME1*	Malic enzyme 1	m	h
	*G6PD*	Glucose-6-phosphate 1-dehydrogenase	m	h
	*PGD*	6-Phosphogluconate dehydrogenase	m	h
Drug transporters	*ABCB6*	ATP-binding cassette, subfamily B, member 6	m	h
	*ABCC1*	ATP-binding cassette, subfamily C, member 1	m	
	*ABCC2*	ATP-binding cassette, subfamily C, member 2	m	h
	*ABCC3*	ATP-binding cassette, subfamily C, member 3	m	h
	*ABCC4*	ATP-binding cassette, subfamily C, member 4	m	
	*ABCC5*	ATP-binding cassette, subfamily C, member 5	m	
Heme and metal metabolism (stress response protein)	*HO-1*	Heme oxygenase-1	m	h
	*FTH1*	Ferritin, heavy polypeptide 1	m	h
	*FTL1*	Ferritin, light polypeptide 1	m	h
	*MT1*	Metallothionein 1	m	h
	*MT2*	Metallothionein 2	m	h
Protein degradation	*UbC*	Ubiquitin C	m	
	*PSMB5*	Proteasome 26S PSMB5 subunit	m	
Lipid metabolism	*ACOT7*	Acetyl-CoA thioesterase 7	m	
	*ACOX1*	Acetyl-CoA oxidase 1	m	
	*LIPH*	Lipase, member H	m	
	*CES1G*	Carboxylesterase 1G	m	

### 3.2. NRF2 as a Multi-organ Protector against Oxidative Damages

Since NRF2 governs the expression of genes associated with xenobiotic metabolism and redox homeostasis, *nrf2* null mice have been relatively more susceptible to acute damages of acetaminophen [87], cigarette smoke [88], and diesel exhaust [89] than wild-type mice. These mutant mice suffered from pulmonary inflammation following ovalbumin sensitization and showed a more severe asthmatic response than wild-type mice [90]. Cytotoxicity by 3-nitropropionic acid (3-NP), an inhibitor of mitochondrial complex II, was enhanced in *nrf2*-deficient primary neurons. In addition, *nrf2*-null mice showed increased lesion volume in the striatum along with impaired rotarod task performance following 3-NP injection [91]. 

The role of NRF2 in renoprotection has been suggested in multiple studies. Female mice with *nrf2* gene ablation displayed a lupus-like autoimmune nephritis at over 60 weeks of age [92]. In a model of ischemia-reperfusion injury, renal function, vascular permeability, and survival of *nrf2*-knockout mice were significantly worse than wild-type mice [93]. Renal damage and interstitial fibrosis by cyclosporin A treatment were relatively higher in *nrf2*-knckout mice [94]. The STZ-induced diabetic nephropathy model revealed that *nrf2*-null mice developed a severe renal injury with greater oxidative DNA damage than wild-type mice [95]. 

Accordantly, there are a number of studies showing favorable effects of NRF2 inducers. Pharmacological intervention of NRF2 activators exerted protective roles against injuries from oxidative stress and inflammation in various* in vitro* and *in vivo* experimental models. Particularly, naturally occurring NRF2 activators have gained great attention: sulfur-containing compounds and phenolic antioxidant compounds are major dietary NRF2 activators [96,97]. In addition to these major groups, indoles, diterpenes, coumarins, and lactones are also regarded as NRF2 inducers [98]. Sulforaphane (SFN) and phenethyl isothiocyanate, which are abundant constituents of cruciferous plants, are well characterized NRF2 activators containing a sulfur motif, which can lead to KEAP1 sulfhydryl modification and ultimately NRF2 activation [97]. Cysteine residues of KEAP1 have an important role in activating NRF2 by SFN. Specifically, modification of Cys151 by SFN treatment is required for liberating NRF2 from KEAP1 [99]. A majority of evidence from *in vitro* and *in vivo* experiments elucidated that SFN increases the expression of phase II detoxifying enzymes and antioxidant proteins via NRF2 signaling [100,101]. In human hepatocarcinoma and prostate cancer cells, expression of NQO1 and GSTs were up-regulated by SFN treatment [102,103]. In an *in vivo* experiment, SFN treatment showed increased expression of NQO1 and GCL in intestines of wild-type mice but not in *nrf2*-null mice [104]. Incubation of phenethyl isothiocyanate also increased NQO1 and UDP-glucuronosyl transferase (UGT) expressions in Hepa1c1c7 cells [105]. Resveratrol, curcumin, cinnamic aldehyde (CA), and quercetin are phenolic antioxidant compounds. As a common chemical property, they have an electrophilic α, β-unsaturated carbonyl moiety called a Michael acceptor. This moiety is highly reactive to the Cys residues of KEAP1, which in turn induces conformational changes of KEAP1 [106,107]. In an early study by Dinkova-Kostova *et al.*, dietary constituent curcumin increased phase II enzyme expression in murine hepatoma cells via arylhydrocarbon (Ah) receptor-independent manner [108]. There has been considerable evidence that people who consumed the foods containing quercetin had a lower risk of cancer than the control group [109]. In subsequent studies, the pharmacological benefits of quercetin were attributed to activation of NRF2 and its target gene expression [110,111].

### 3.3. NRF2 as Anti-inflammatory Modulator

There are notable reports showing reciprocal regulation of NRF2 and NFκB, suggesting an anti-inflammatory role of NRF2. For the activation of NF-κB, IκB, a cytosolic inhibitor protein of NF-κB, has to be phosphorylated by IκB kinase, and this leads to the degradation of IκB and nuclear translocation of NF-κB [112]. In this serial event, NRF2 is known to control NF-κB levels through suppression of IκB phosphorylation [113]. Mouse embryonic fibroblasts (MEF) from *nrf2* null mice displayed a higher level of phosphorylated IκB than wild-type MEF [114], and these mutant cells possessed diminished levels of NF-κB subunit p50 and p65 [115]. In spinal cord injury mice, *nrf2* knockout mice showed elevated NF-κB activity and TNFα expression [116]. Additionally, there have been reports on the role of NRF2 target proteins for NF-κB inhibition. Overexpression of HO-1 led to stabilization of IκB, resulting in NF-κB inhibition. Consistently, the inhibition of HO-1 activity suppressed p65 activity [117]. Overexpression of NRF2 or NQO1 repressed LPS-inducible expressions of TNFα and IL-1β in human monocytes [118]. Similarly, SFN treatment decreased DNA binding activity of NF-κB without affecting IκB levels in murine macrophages [119]. NRF2 inducer curcumin inhibited NF-κB activation through the down-regulation of IκB kinase [120]. A potent NRF2 inducer triterpenoid bardoxolone methyl directly blocked IκB kinase by interacting with Cys179 of the IκB kinase [121]. 

## 4. Role of the NRF2 System in CKD

Today increasing evidence supports the critical role of NRF2 in kidney pathology. In lupus nephritis, oxidative stress and inflammation have been suggested as core pathologic components: glomerular deposition of immune complex can bring about oxidative damage, leading to glomerular injuries [122]. Earlier, it was shown that female mice with *nrf2* gene deletion developed lupus-like autoimmune nephritis [92]. A recent study by Jiang *et al*. [123] demonstrated that *nrf2*-deficient mice developed a greater degree of renal damages in a pristine-induced lupus nephritis model. In addition, administration of epigallocatechin-3-gallate prevented development of nephritis in spontaneously lupus-prone mice and this protection was mediated by NRF2 signaling [124]. Similarly, curcumin and bardoxolone methyl attenuated lupus nephritis in this lupus mouse model [125,126].

Experimental evidence showing the involvement of NRF2 in diabetic nephropathy was provided by multiple groups. Yoh *et al.* demonstrated a protective role of NRF2 in diabetic complications [127]. When diabetes was induced by a single injection of STZ, *nrf2*-null mice developed renal impairments earlier and suffered more severe mesangial injuries than wild-type mice. Similarly, in a study by Jiang *et al*. [95], when ROS production increased, oxidative DNA damage and renal injury worsened in STZ-treated *nrf2* null mice compared to wild-type mice. In another study, these mutant mice showed exacerbated acute renal injuries following ischemia-reperfusion or cisplatin treatment. On the other hand, the administration of GSH or its precursor N-acetylcysteine could improve renal function in these mice [93]. Multiple *in vitro* studies also support the protective effects of NRF2 on renal pathology. TGFβ-stimulated ECM production was exacerbated in *NRF2*-silenced renal tubular epithelial cells, whereas TGFβ signaling and ECM expression were largely attenuated in *KEAP1*-silenced cells [128,129].

The study by Kim *et al.* [130], which used male Sprague-Dawley rats, was the first report to show the involvement of NRF2 signaling in an experimental model of 5/6 nephrectomy CKD. In this model, the remnant kidney exhibited GSH depletion, lipid peroxidation, NF-κB stimulation and NADPH oxidase activation. Notably, NRF2 activity was significantly diminished at 12 weeks in the remnant kidney, leading to a decrease in CAT, SOD, HO-1 and NQO1. In a study with Imai rats that is a model of spontaneous focal glomerulosclerosis, nuclear NRF2 levels and its target gene expressions were markedly reduced in the kidney in spite of the presence of a severe oxidative and inflammatory pathologic environment [26]. In a tubulointerstitial nephropathy model using adenine treatment, NRF2 activity was impaired and the expressions of CAT, HO-1 and GCL were repressed [131].

The above mentioned reports provide evidence for the necessity of NRF2 in animal models of CKD, and raises a possibility that dysregulation of NRF2 signaling may be involved in human CKD pathology. Indeed, lines of evidence show that the expression of several NRF2 target genes is diminished in kidneys from CKD patients. Activities of GPx and GSR were significantly decreased in T2DM patients with or without nephropathy. Moreover, repression of GPx and GSR was more severe in diabetic patients with nephropathy compared to patients without nephropathy [132]. According to Crawford *et al*. [133], CKD patients possessed lower levels of blood monocyte GPx and red blood cell CAT activities than controls. It was also notable that the single nucleotide polymorphism of GPx was more prevalent in CKD patients, suggesting the role of GPx in CKD pathology. A study by Puchades *et al.* [134] showed that levels of oxidized molecules such as protein carbonyls and 8-oxo-dG, and NRF2 target gene expression are increased in CKD patients with hemodialysis or peritoneal dialysis. Results showed that levels of oxidized molecules were significantly high, and enzyme activities of GPx, GSR, CAT and SOD were markedly diminished in these patients compared to the control group. 

## 5. Naturally Occurring NRF2 Activators and CKD

Recently, many groups have demonstrated the beneficial effects of NRF2 on CKD. A study by Li *et al*. [135] elucidated how the increase of NRF2 target HO-1 and GCL is required in high glucose conditions to counteract high glucose-induced ROS increase and resultant oxidative damages in cultured mesangial cells. Due to protective effects, *nrf2*-null mice were vulnerable to STZ-induced nephropathy, characterized by severe renal dysfunction and oxidative injuries [95,127]. Therefore, experimental conditions with NRF2 activation *in vitro* and *in vivo* could provide protection against CKD. It was shown that incubation of human skin fibroblasts with proteasome inhibitor MG132 could induce nuclear translocation of NRF2 and increase target gene expression, suggesting that proteasome inhibitor treatment can activate NRF2 signaling [136]. When Sprague-Dawley rats were administered with low doses of MG132 for 12 weeks, renal mRNA levels for SOD1, CAT, and GPx increased and STZ-induced DN significantly improved [137]. A study by Cui *et al*. [138] used OVE26 mice as a model of transgenic type 1 diabetic mice. When a low dose of MG132 was administered to OVE26 for 3 months, there were significant improvements in renal structure alterations and kidney function.

In contrast, indoxyl sulfate (IS), one of the uremic toxins casing endothelial dysfunction and nephrotoxicity [139,140], was shown to repress renal expression of NRF2. A research by Bolati *et al*. [141] elucidated that IS administration in rats reduced the level of NRF2 and its target gene expression in the kidney, thereby increasing the renal level of 8-oxo-dG. However, treatment with AST-120, an oral sorbent lowering serum IS level, recovered NRF2 expression in CKD rat kidneys and consequently diminished ROS production.

On the basis of these reports, the positive relationship between NRF2 and CKD protection gained attention, leading researchers to make an effort to develop NRF2 activators as a novel therapeutic strategy for CKD protection/retardation. In particular, there have been extensive investigations on naturally occurring NRF2 activators for their potential efficacy in CKD protection. Here we review current findings of the role of NRF2 activating SFN, resveratrol, curcumin, and CA (chemical structures are shown in Table 3) in different animal CKD models.

### 5.1. SFN

SFN is abundant in cruciferous vegetables such as broccoli, brussels sprouts and cabbages [142]. It has long been recognized that SFN has potent chemopreventive efficacy in various animal carcinogenesis models and it had been accepted that the induction of phase II detoxifying enzymes is an underlying mechanism of SFN effect [143]. In addition to cancer prevention, accumulating evidence indicates that SFN effectively protects multiple organs from oxidative injuries. SFN treatment in cultured cardiomyocytes reduced ROS and DNA fragmentation [144] and the administration of SFN-containing broccoli in rats protected their hearts from ischemia-reperfusion injuries [145]. Animals fed SFN displayed reduced production of inflammation markers such as IL-6 and TNFα in response to LPS treatment in the brain [146]. Now, it has been firmly established that these multi-organ protective effects of SFN are attributed to activation of NRF2 signaling [147,148].

**Table 3:** Chemical structures of NRF2 activators.

NRF2 Activators	Chemical Structure
Sulforaphane (SFN)	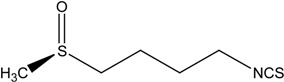
Resveratrol	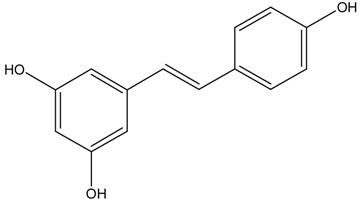
Curcumin	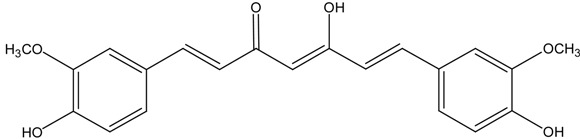
Cinnamic aldehyde (CA)	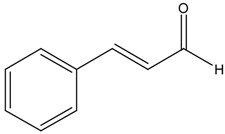
Bardoxolone methyl	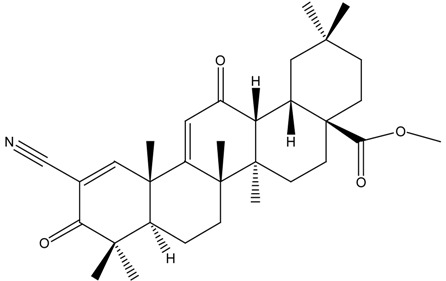

The renoprotective effects of SFN have been evidenced in several *in vivo* studies. SFN administration protected kidneys from cisplatin and ischemia-reperfusion challenge and these protections were mediated through NRF2 activation [149,150,151]. A study by Zheng *et al.* [152] demonstrated that, in a STZ-induced diabetic mouse model, SFN treatment (started 2 weeks after STZ injection) noticeably improved metabolic dysfunction associated with diabetes, albuminuria and glomerular sclerosis. These beneficial effects of SFN were not examined in *nrf2* deficient mice, indicating that NRF2 is a molecular target of SFN. This study further revealed that SFN attenuates high glucose-induced mesangial cell hypertrophy by NRF2-mediated TGFβ signaling repression. In a study by another group, SFN administration (0.5 mg/kg) for 3 months prevented STZ-induced DN: the albumin-to-creatinine ratio (ACR) and renal fibrosis were alleviated by SFN [153]. In addition, *in vitro* experiments using the human renal tubular KH11 cell line showed that oxidative damages by mannitol or high glucose plus palmitate were attenuated by SFN incubation; however protection was lost following *NRF2* siRNA introduction. Beneficial effects of SFN were also demonstrated in the UUO model. In rats, UUO induced mitochondrial stress and subsequent apoptosis by decreasing nuclear NRF2 levels, whereas SFN treatment reversed NRF2 levels and modulated mitochondrial-stress damages and renal fibrosis [154]. An additional study with UUO showed that structural renal damages by UUO was remarkably improved after SFN treatment in rats [155].

### 5.2. Resveratrol

Resveratrol (3,5,4'-trihydroxystilbene) is a polyphenolic phytoalexin which is found in many plant species, including grapes and berries [156]. Initially, resveratrol gained attention for its cardioprotective effects when this compound, a key ingredient of red wine, was implicated in the French Paradox: the low incidence of cardiovascular diseases in the French population [157]. After this, numerous biological activities of resveratrol were identified. Resveratrol has been associated with multiple pharmacological health benefits that stem from its antioxidant [158], anti-inflammatory [159], anti-mutagenic [160], and anticancer properties [161]. This phytochemical exerts antioxidant effects by directly scavenging ROS or by increasing antioxidant enzyme expression through NRF2, AP-1 and forkhead box O (FOXO) [162,163,164]. In addition, resveratrol activates silent information regulator 1 (SIRT1), an NAD^+^-dependent deacetylase, via AMP-activated protein kinase (AMPK) activation [165,166]. Through these signaling alterations, resveratrol exhibits renoprotective effects in various animal models. In rat renal mesangial cells, resveratrol incubation decreased high concentration glucose-induced cell proliferation and fibronectin expression through inhibition of NF-κB/NADPH oxidase pathway [167]. Kitada *et al.* demonstrated the effect of resveratrol in a diabetic nephropathy model of *db/db* mice: resveratrol supplementation (400 mg/kg) attenuated albuminuria, urinary 8-oxo-dG excretion, and histological changes of kidney in *db/db* mice via AMPK-SIRT1-independent manner [162]. A study by Kim *et al.* confirmed beneficial effects of resveratrol in *db/db* mice [165]. Resveratrol treatment was started at the age of 8 weeks (maintained for 12 weeks) and it inhibited renal lipotoxicity and oxidative stress by enhancing AMPK-SIRT1-PGC1α signaling. Sharma *et al*. demonstrated a protective effect of resveratrol in a STZ diabetic model. The administration of resveratrol in STZ-treated rats improved renal dysfunction by reducing albuminuria and significantly diminishing oxidative stress markers such as MDA [168]. Resveratrol was also effective in preventing renal fibrosis in a UUO model: administration of resveratrol inhibited Smad3 acetylation, resulting in TGFβ signaling repression [169,170]. Together with these molecular targets, recent studies show that resveratrol affects NRF2 signaling in the kidney. Palsamy *et al.* reported that resveratrol treatment (5 mg/kg) for 30 days diminished creatinine clearance to the normal level in STZ/nicotinamide-induced diabetic rats [171]. Resveratrol also restrained lipid peroxidation and protein carbonyl levels in STZ rats. In particular, this study demonstrated that decreased expression of NRF2, GCS, GST and HO-1 in the STZ-diabetic kidney was normalized by resveratrol treatment. An *in vitro* experiment by Huang *et al.* showed that activation of SIRT1 by resveratrol leads to NRF2 increase in glomerular mesangial cells. Eventually, this event prevented AGE-induced TGFβ and fibronectin expressions [172].

Although there is no direct clinical evidence showing protective effects of resveratrol in CKD patients, several studies imply its beneficial effects on human subjects. Resveratrol supplementation in T2DM patients significantly reduced insulin resistance and urinary excretion of ortho-tyrosine levels, which is a marker of oxidative stress [173]. Moreover, dietary supplementation with red grape juice exerted antioxidative and anti-inflammatory efficacies in hemodialysis patients [174,175].

### 5.3. Curcumin

Curcumin, a phenolic compound derived from the hebaceous plant *Curcuma longa*, has been used as a traditional medicine for a long time [176]. Numerous studies have shown that curcumin has various biological and pharmacological functions; antioxidant [177], anti-inflammatory [178], anti-bacterial [179], anti-proliferative [180], hepatoprotective [181], cardioprotective [182] and neuroprotective activities [183]. In particular, curcumin was shown to improve diabetic symptoms and protect DN through multiple molecular targets [184]. In STZ diabetic rats, curcumin treatment (15 and 30 mg/kg) for 2 weeks after STZ injection ameliorated the increase of creatinine and urea clearance, proteinuria and oxidative stress markers [185]. In another similar experimental design, curcumin (100 mg/kg) treatment for 8 weeks significantly reversed all of abnormalities derived from STZ, including proteinuria, reduced creatinine clearance, and increased blood urea nitrogen [186]. Several signaling molecular targets have been suggested as underlying mechanisms of renoprotection of curcumin. In a study by Soetikno *et al*., normalization of renal dysfunction and lipid peroxidation in STZ rats was associated with inhibition of PKC-α and PKC-β1 by curcumin [187]. Chiu *et al*. demonstrated that renal oxidative stress, mesangial proliferation, and activation of histone acetyltransferase p300 and NF-κB by STZ were prevented by curcumin treatment [188]. Curcumin (100 mg/kg) administration in STZ diabetic animals markedly decreased infiltration of renal macrophages and renal production of proinflammatory cytokines such as TNF-α and IL-1β along with NFκB inhibition. In cultured glomerular mesangial cells, curcumin incubaton inhibited high concentration glucose-induced activation of activator protein-1 (AP-1) and sphingosine kinase 1 (SphK1) signaling, resulting in the supression of SphK1-regulated fibronectin and TGFβ overexpression [186,189]. In 5/6 nephrectomized rats, Ghosh *et al*. found that curcumin administration (75 mg/kg) for 8 weeks improved renal dysfunction and inhibited NF-κB activation and macrophage infiltration in the kidney [190].

Due to the poor bioavailability of curcumin, efforts have been made to improve the pharmacokinetic profile of this compound. A curcumin derivative B06 was developed for this reason and was proved to be effective in improving renal function and macrophage infiltration in diabetic rat kidneys [191]. B06 (0.2 mg/kg, 6 weeks) also inhibited pro-inflammatory cytokine production through the repression of c-Jun N-terminal kinase/NF-κB signaling. Another renoprotective curcumin derivative C66 was associated with the inactivation of mitogen-activated protein kinase (MAPK) pathway and the down-regulation of ACE/angiotensin II system [192].

Recent evidence supports the strong involvement of NRF2 signaling in renoprotective effects of curcumin. In a high-fat diet (HFD) mouse model, He *et al*. described that the short-term administration of curcumin (15 days) increases NRF2 signaling and diminishes lipid peroxide makers in the muscle, improving insulin tolerance, implying the beneficial effects of curcumin-NRF2 on diabetes [193]. A study by Soetikno *et al*. used a 5/6 nephrectomized rat model to demonstrate that curcumin treatment (75 mg/kg) prevents the decrease of NRF2 and HO-1 expressions in the remnant kidney along with the amelioration of renal dysfunction [194]. Similarly, other groups reported that NRF2 activation by curcumin is necessary for prevention of hemodynamic changes, glomerular hypertension, hyperfiltration, and antioxidant enzyme decrease in 5/6 nephrectomized rats [195,196]. 

Several clinical trials have evaluated the potential efficacy of curcumin in diabetes and DN. Usharani *et al*. showed that curcumin treatment (300 mg, twice a day for 8 weeks) in patients with T2DM significantly enhanced endothelial function and decreased oxidative stress and inflammatory markers [197]. A randomized, double-blind clinical trial by Chuengsamarn *et al*. reported that curcumin administration (1.5 g per day, 9 months) in pre-diabetic population was beneficial in preventing T2DM development, which was assessed by β-cell function, insulin resistance, and serum cytokine levels [198]. These studies allude to the potential benefit of curcumin in human DN and one clinical trial has been conducted to investigate this possibility. In a randomized, double-blind and placebo-controlled study by Khajehdehi *et al*. [199], patients with DN were received by turmeric (22.1 mg of curcumin, three times a day) for 2 months and the levels of TGFβ and pro-inflammatory cytokines were evaluated. Results indicate that serum levels of TGFβ and IL-8, and urinary levels of IL-8 were significantly decreased after curcumin supplementation when compared to the pre-supplementation group. In addition, proteinuria in DN patients was effectively improved by this supplementation without adverse effects. 

### 5.4. CA

CA is a major compound isolated from *Cinnamomum cassia* [200]. Various lines of evidence indicate that CA has chemopreventive efficacy [201], anti-bacterial activities [200] and anti-inflammatory effects [202]. In addition, as a reactive Michael acceptor-containing chemical, CA is a potent NRF2 activator that induces the expression of its target genes [203]. There are a number of studies that evaluated renoprotective effects of CA. Zheng *et al*. elucidated that CA can improve diabetic-induced renal injuries [152]. CA treatment was able to ameliorate albuminuria and creatinine clearance in STZ-induced diabetic mice. While CA increased levels of NRF2 and its target genes NQO-1 and GCL, and suppressed TGF-β1 signaling in the kidneys of these diabetic mice, the treatment did not show any protective effect in *nrf2*-deficient mice, confirming the critical role of NRF2 signaling in DN protection. A study by Chao *et al*. [204] also showed that CA administration reduces high concentration glucose-induced cell proliferation and hypertrophy in renal interstitial fibroblast cells. Administration of cinnamon oil in alloxan-treated animals decreased fasting glucose levels and attenuated alloxan-induced DN [205].

## 6. Experience and Promise from Bardoxolone Methyl for CKD Management

Bardoxolone methyl (methyl-2-cyano-3,12-dioxooleano-1,9(11)dien-28-oate, CDDO-Me) is described as a synthetic triterpenoid, derived from the natural product oleanolic acid. This antioxidant inflammation modulator is known as one of most potent activators of NRF2 [206,207,208]. A study by Dinkova-Kostova *et al*. [206], used a series of synthetic triterpenoid analogs and demonstrated that phase II enzyme response and inflammation blocking effects are dependent on the presence of Michael reaction center. Further, a recent study provided direct biochemical evidence that bardoxolone methyl binds to the Cys151 of the BTB domain of KEAP1 forming an adduct which inhibits the association of Cul3 to KEAP1, resulting in NRF2 activation [209]. In addition, bardoxolone methyl binds to the Cys179 residue in IκB kinase IIKKβ protein, resulting in the inhibition of NFκB activation and pro-inflammatory cytokine production [121].

In animal experiments, treatment with bardoxolone methyl protected kidneys from acute injuries by elevating NRF2 target genes. Coordinated induction of NRF2 target genes in bardoxolone-treated kidneys ameliorated ischemia-reperfusion renal injuries [210]. Nephrotoxicity by iron nitrilotriacetate (FeNTA) was more severe in *nrf2* null mice, and triterpenoid treatment protected FeNTA-induced renal injury through NRF2 activation [211]. Moreover, when STZ-induced diabetic mice were administered with a derivative of bardoxolone methyl, diabetes-associated atherosclerosis was reduced and diabetic kidney injury improved [212]. Another synthetic triterpenoid CDDO-dhTFEA could restore NRF2 decrease in rat CKD by 5/6 nephrectomy, and attenuated kidney inflammation and fibrosis [213].

Potential benefits of bardoxolone methyl in human kidney dysfunction have been recognized in cancer patients treated with this drug. The first Phase I clinical trial showed that bardoxolone treatment in patients with lymphomas and advanced solid tumors increased NQO1 mRNA in peripheral blood mononuclear cells and enhanced GFR [214]. After this intriguing observation, bardoxolone methyl was applied in Phase 2 clinical trial for patients with diabetes and CKD. An exploratory multi-center, opens-label, randomized clinical trial was performed to assess the renoprotective effect and safety of bardoxolone methyl [215]. Twenty patients with T2DM and CKD (stage 3 to 4) took 25 mg bardoxolone methyl for 28 days, then 75 mg for further 28 days. About 75% of patients took antihypertensive medicines, statins, and ACE inhibitors or ARBs, and most patients had significant albuminuria. Administration of bardoxolone showed a significant increase in GFR (increase of 2.8 mL/min/1.73 m^2^ at 4 weeks; increase of 7.2 mL/min/1.73 m^2^ at 8 weeks), a decrease in serum creatinine and blood urea nitrogen, and an increase of creatinine clearance. This study brought to light a therapeutic potential of bardoxolone methyl in CKD to light. However, there are some important limitations in this study such as a lack of a placebo control group and short term of treatment.

A large Phase 2b clinical trial (BEAM study) was prepared to confirm the effect of bardoxolone methyl in CKD patients [216]. In this double blind, randomized, placebo-controlled trial, 227 patients with diabetes and CKD participated. They received placebo or bardoxolone methyl with one of three dosage levels: 25, 75 or 150 mg. Bardoxolone methyl administration improved GFR within 4 weeks after treatment initiation of treatment and these improvements persisted for 52 weeks. In addition, adverse events were moderate and manageable, but muscle spasms and a mild increase in alanine aminotransferase levels were common. After this optimistic result, the BEACON trial, a multi-national, double-blind, placebo-controlled Phase 3 study began in 2011 [217]. A total of 2,185 patients with T2DM and stage 4 CKD were randomized and assigned a treatment of either 20 mg/day bardoxolone or placebo. This trial, however, was terminated prematurely in October 2012 by the Independent Data Monitoring Committee due to adverse events and mortality rates. Ninety six patients in the bardoxolone methyl group were hospitalized or dead due to heart failure, whereas in the placebo group, merely 55 cases were reported. In remaining patients, blood pressure, estimated GFR, and the urinary albumin-to-creatinine ratio increased significantly compared to those of the placebo group.

## 7. Conclusions

**Figure 2:**
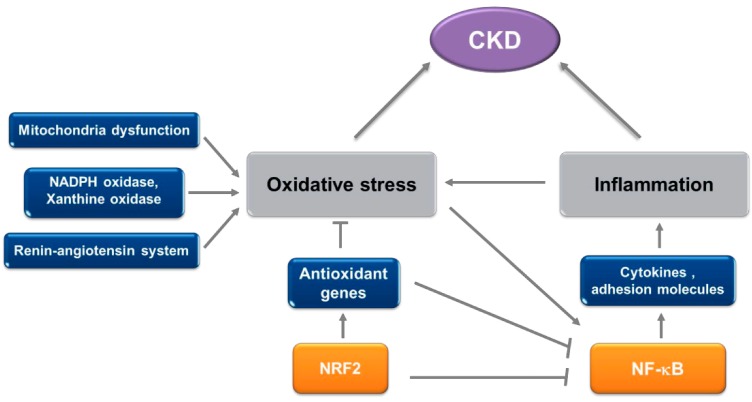
Factors involved in the progression of kidney dysfunction in CKD and the role of NRF2. In CKD, the alteration of mitochondrial function and the activation of ROS-generating enzymes such as NADPH oxidase and xanthine oxidase participate in aggravated oxidative stress condition in the kidney. The activation of the renin-angiotensin system is another important contributing factor. In addition, oxidative stress triggers NF-κB activation and enhances inflammatory response, which is an important pathologic component of CKD. NRF2 provides renal cells with antioxidant potential by up-regulating an array of genes and consequently attenuates the production of pro-inflammatory cytokines and adhesion molecules.

The failure of the bardoxolone methyl clinical study suggests the importance of animal experiments to identify precise modes of action of candidate drugs before applying them to a clinical setting; however, there still remains a possibility that NRF2 activators can be beneficial in managing CKD. As illustrated in Figure 2, there is increasing evidence that oxidative stress and inflammation are integrated contributing factors to CKD progression. Recent extensive studies have established the role of NRF2 signaling in renal protection against oxidative damage, and in modulation of inflammatory response. In addition, due to their traditional application for thousands years, plant-derived NRF2 activators such as resveratrol and curcumin have demonstrated their safety and health benefits in human subjects. Therefore, the utilization of naturally occurring NRF2 activators which share antioxidant and anti-inflammatory efficacies in CKD may be a promising strategy to ameliorate or retard kidney dysfunction.

## Abbreviation

CKD: chronic kidney disease
T2DM: type 2 diabetes mellitus
DN: diabetic nephropathy
GFR: glomerular filtration rate
ECM: extracellular matrix
UUO: unilateral ureteral obstruction
RAS: renin-angiotensin system
ACE: angiotensin-converting enzyme
STZ: streptozotocin
AGE: advanced glycation end products
ROS: reactive oxygen species
8-oxo-dG: 8-oxo-7,8-dihydro-2'-deoxyguanosine
GSH: glutathione
GCL: γ-glutamate cysteine ligase
SOD: superoxide dismutase
GPx: glutathione peroxidase
NQO1: NAD(P)H quinone oxidoreductase-1
GST: glutathione *S-*transferase
HO-1: heme oxygenase-1
UGT: UDP-glucuronosyl transferase
TNFα: tumor necrosis factor-α
IL: interleukin
MDA: malondialdehyde
SFN: sulforaphane
CA: cinnamic aldehyde
